# Fronto-temporal interactions are functionally relevant for semantic control in language processing

**DOI:** 10.1371/journal.pone.0177753

**Published:** 2017-05-15

**Authors:** Max Wawrzyniak, Felix Hoffstaedter, Julian Klingbeil, Anika Stockert, Katrin Wrede, Gesa Hartwigsen, Simon B. Eickhoff, Joseph Classen, Dorothee Saur

**Affiliations:** 1 Language and Aphasia Laboratory, Department of Neurology, University of Leipzig, Leipzig, Germany; 2 Institute of Clinical Neuroscience and Medical Psychology, Medical Faculty, Heinrich Heine University Düsseldorf, Düsseldorf, Germany; 3 Institute of Neuroscience and Medicine (INM-1), Research Center Jülich, Jülich, Germany; 4 Institute for Systems Neuroscience, Medical Faculty, Heinrich Heine University Düsseldorf, Düsseldorf, Germany; 5 Department of Neuropsychology, Max Planck Institute for Human Cognitive and Brain Sciences Leipzig, Leipzig, Germany; 6 Human Cortical Physiology and Motor Control Laboratory, Department of Neurology, University of Leipzig, Leipzig, Germany; University of Cambridge, UNITED KINGDOM

## Abstract

Semantic cognition, i.e. processing of meaning is based on semantic representations and their controlled retrieval. Semantic control has been shown to be implemented in a network that consists of left inferior frontal (IFG), and anterior and posterior middle temporal gyri (a/pMTG). We aimed to disrupt semantic control processes with continuous theta burst stimulation (cTBS) over left IFG and pMTG and to study whether behavioral effects are moderated by induced alterations in resting-state functional connectivity. To this end, we applied real cTBS over left IFG and left pMTG as well as sham stimulation on 20 healthy participants in a within-subject design. Stimulation was followed by resting-state functional magnetic resonance imaging and a semantic priming paradigm. Resting-state functional connectivity of regions of interest in left IFG, pMTG and aMTG revealed highly interconnected left-lateralized fronto-temporal networks representing the semantic system. We did not find any significant direct modulation of either task performance or resting-state functional connectivity by effective cTBS. However, after sham cTBS, functional connectivity between IFG and pMTG correlated with task performance under high semantic control demands in the semantic priming paradigm. These findings provide evidence for the functional relevance of interactions between IFG and pMTG for semantic control processes. This interaction was functionally less relevant after cTBS over aIFG which might be interpretable in terms of an indirect disruptive effect of cTBS.

## Introduction

Semantic cognition, i.e. processing of meaning is a central function in language and communication. Previous studies suggested that two distinct components of semantic cognition can be segregated, semantic representation and semantic control, which have different neural underpinnings [1,2]. Semantic representations encode multimodal concepts which span different items and contexts. They are thought to be implemented in distributed modality specific cortices which interact with a transmodal hub located bilaterally in the anterior temporal lobe [3]. On the other hand, semantic control is necessary to specifically retrieve context-relevant and task-appropriate semantic information from the representational system especially when unusual, uncharacteristic or anomalous meanings need to be accessed or when dominant meanings need to be suppressed. For example, a very salient feature of the concept “piano” is that it is a musical instrument, but in the context of moving, the most important semantic aspect may be its weight [4]. Increasing semantic control demands are thereby associated with higher task-related activity in left inferior frontal gyrus (IFG), angular gyrus, dorsomedial prefrontal cortex and posterior middle temporal gyrus (pMTG) [5–7]. The latter region is of special interest because it might form an interface between networks which underlie automatic and controlled semantic retrieval [8]. Previous transcranial magnetic stimulation (TMS) studies confirmed a causal relationship between semantic control and both IFG and pMTG: semantic judgements were delayed and less accurate after inhibitory TMS over IFG or pMTG only for trials with high semantic control demands (i.e. weakly associated cue and target words) [9,10]. Another study combining inhibitory rTMS and fMRI with a similar semantic priming paradigm unexpectedly reported decreased activity in bilateral IFG and dorsomedial prefrontal cortex exclusively for trials with low control demands while reaction times remained unaffected [11]. The fact that activity associated with high control demand trials was not suppressed due to rTMS might reflect the specific role of these regions for semantic control.

In language comprehension, predictions are formed about upcoming words depending on the prior context [12–15]. It is assumed that correct predictions might facilitate access to semantic representations and thus promote integration of a word in the given context [16,17]. False predictions on the other hand require additional integration efforts which can be viewed as a situation with high semantic control demands because contextually (spuriously) preactivated representations need to be inhibited [18]. For example, the context of “the pilot flies the …” might lead to the prediction and accompanying preactivation of the concept “plane” which then needs to be suppressed when the actual sentence ending is unexpected (e.g. “kite”) or even incorrect (e.g. “radiator”). Behaviorally, this is reflected by increased reaction times for unexpected or incorrect in relation to expected words in naming or lexical decision tasks [13]. Functional magnetic resonance imaging (fMRI) studies have also shown that such semantic violations are accompanied by increased activity in semantic control networks in left anterior IFG (aIFG), pMTG and additionally anterior middle temporal gyrus (aMTG) [19,20,15,21].

Resting-state fMRI is a tool to map functional networks based on the temporal similarity of slow intrinsic blood oxygenation level dependent (BOLD) signal fluctuations during wakeful rest [22,23]. Regions with significantly similar BOLD signals at rest are assumed to be functionally connected. Functional connectivity at rest is thereby thought to facilitate coordinated task-related activity. This is reflected in similar network mappings at rest and for corresponding tasks in fMRI investigations [24]. It has further been shown that such measures of resting-state functional connectivity are of functional relevance for language processing since they correlate with behavioral performance [25–32]. Previous studies have revealed highly interconnected, left-lateralized fronto-temporal networks for general language [33–35] as well as speech production [36] and comprehension [37] processes during rest. A recent study by Wei and colleagues demonstrated that regional BOLD signal amplitudes of left pMTG during rest are associated with general semantic processing. Furthermore, functional resting-state connectivity of this region with left IFG, other parts of the MTG and dorsomedial prefrontal cortex also predicted performance in semantic processing [28]. Less is known about the functional relevance of resting-state functional connectivity for more specific semantic operations like semantic control. Besides, while some studies have documented effects of transcranial magnetic stimulation (TMS) on resting-state networks [38–47], few studies have investigated specifically whether behavioral alterations due to TMS are also reflected in changes of resting-state functional connectivity. Recent work by Wang et al. could prove such a mechanism for cortical-hippocampal networks and associative memory: High-frequency repetitive TMS delivered over left lateral parietal cortex for five consecutive days led to increased resting-state functional connectivity between hippocampus and multiple cortical sites which correlated with an increased associative memory performance [39].

In the present study, we characterized the semantic network of 20 healthy subjects based on resting-state functional connectivity of three regions of interest (ROIs) which were associated with semantic control processes in a prior study, i.e. left aIFG, aMTG and pMTG [21]. We further investigated the relevance of functional connectivity within this network for semantic control processes and finally aimed to modulate this network with the application of continuous theta burst stimulation (cTBS) over left aIFG and pMTG. We hypothesized that connectivity patterns of the three ROIs would reveal the semantic system found in previous task-based fMRI studies and that the strength of functional coupling between these nodes at rest would predict performance in a semantic priming paradigm with varying semantic control demands. We further hypothesized that cTBS over left aIFG and/or pMTG would interfere with semantic processing in trials with high semantic control demands and that this effect would be mediated by cTBS induced changes in functional connectivity.

## Materials and methods

### Experimental design and procedure

**Fig 1:**
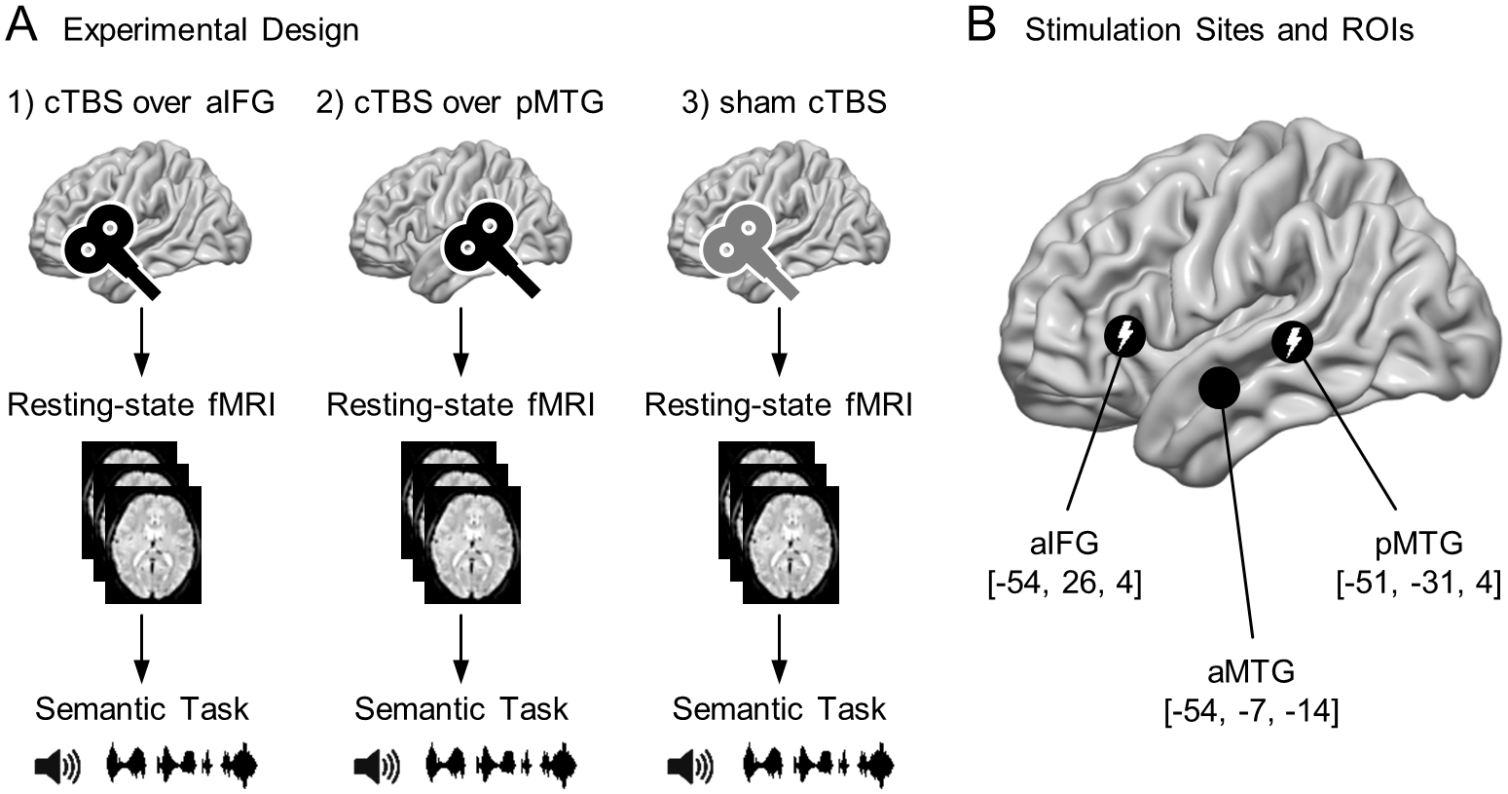
Experimental design with stimulation sites, regions of interest. (A) Experimental design: sham controlled crossover within-subject design with three sessions consisting of cTBS over left (1) aIFG or (2) pMTG or (3) sham stimulation over aIFG or pMTG followed by resting-state functional magnetic resonance imaging and a semantic task. (B) Regions which have been shown to be associated with semantic control processes in a prior study [21] are employed as cTBS stimulation sites (left aIFG and pMTG) and regions of interest (left aIFG, aMTG and pMTG) for resting-state functional connectivity analysis. Anatomical labels according to AAL2-Toolbox for SPM12. Numbers refer to coordinates in MNI space. Brain renderings show the left hemisphere.

The experiment (see Fig 1A) employed a sham controlled crossover within-subject design. Twenty healthy participants underwent neuronavigated cTBS followed by 6 minutes of resting-state fMRI (8.9 ± 0.4 minutes after stimulation; mean ± standard deviation) and a subsequent lexical decision task under varying semantic control demands. In three separate sessions, either real cTBS or sham cTBS was applied over left aIFG or pMTG. All sessions were at least 7 days apart and their order was randomized and counterbalanced across subjects to the best possible degree. A high resolution T1 weighted structural MRI was acquired for individually neuronavigated cTBS on a separate day prior to the first session if not available from prior studies.

### Participants

Twenty adults (10 female) aged 25.1 ± 2.5 years (mean ± standard deviation) participated in the study. All were right handed as given by a mean laterality index of 93%, range 73–100% (Oldfield, 1971). Participants were German native speakers, had no history of neurological or psychiatric illness and no contraindications to MRI scanning or TMS. The study was approved by the ethics committee of the University of Leipzig. All participants gave written informed consent prior to the study.

### Transcranial magnetic stimulation

Transcranial magnetic stimulation was applied as neuronavigated cTBS over left aIFG, pMTG or as sham stimulation over either region prior to resting-state fMRI. The coil was placed tangentially on the head with the handle pointing back- and downwards at 45° in projection to the sagittal plane. Stimulation consisted of 600 biphasic TMS pulses (3 pulses at a rate of 50 Hz every 200 ms) at 80% of the individual active motor threshold (AMT) resulting in a total duration of 40 seconds [48]. The AMT was defined as the stimulation intensity (over the left motor hotspot) which had a 50% chance to produce a motor evoked potential (MEP) of > 200 μV in the tonically active (as defined by amplitudes of about 150 μV in the electromyography) right first dorsal interosseous muscle. This was assessed with an adaptive PEST (parameter estimation by sequential testing) procedure [49] as implemented in the software “Adaptive PEST for TMS” (http://clinicalresearcher.org/software.htm). Because the AMT is known to be variable across individuals but rather stable across time [50], the AMT was determined only once before the first cTBS session and then used for all subsequent sessions.

Stimulation was applied using a MagPro X100 (MagVenture, Medtronic, USA) stimulator and a figure of eight coil (diameter 75 mm, CB-B60, MagVenture). For sham stimulation a similar coil with additional effective magnetic shielding (MCF-P-B65, MagVenture) providing the same acoustic (‘click’) sensation without actual magnetic stimulation was used. Electromyography was derived with Ag/AgCl electrodes, an analog-digital converter “Power 1401 Mk II” and an amplifier “1902” manufactured by Cambridge Electronic Design (UK). Neuronavigation was used in order to ensure best spatial accuracy, which is about 5–8 mm [51,52]. Peak coordinates for left aIFG and pMTG from a prior study [21] (see Fig 1B) were transformed to the individual T1 weighted images with SPM8 (Wellcome Trust Centre for Neuroimaging). Brainsight software (version 1.7.6, Rogue Research Inc., Canada) and an infrared tracking system (Polaris Spectra, Northern Digital Inc., Canada) with trackers fixed to the subjects head and the TMS coil were then used to coregister the subjects head and his/her T1 weighted image based on 7 anatomical landmarks allowing for real-time neuronavigation.

### Magnetic resonance imaging

A 3 Tesla Siemens Verio (Siemens Erlangen, Germany) scanner was used to obtain a T1 weighted MPRAGE (Magnetization Prepared Rapid Gradient Echo) sequence (32-channel head coil, TR/TE: 1300/3.5 ms, flip angle: 10°, voxel size: 1 mm isotropic) for each participant. Resting-state scanning was performed about 9 minutes after cTBS as T2*-weighted EPI (Echo Planar Imaging) sequence (12-channel head coil, TR/TE: 2000/30 ms, flip angle: 90°, voxel size: 3 mm isotropic, slice gap: 1 mm, FOV: 192x192x119 mm, 180 scans total ≙ 6 minutes) while participants had their eyes closed but stayed awake as confirmed by post-scan debriefing.

### Semantic task and stimuli

Subjects performed a well-established semantic priming paradigm with a lexical decision task which implicitly tests lexico-semantic performance under different semantic control demands [20,21]. In short German sentences (1.2–2.4 s) the final word integrated differently well into the prior semantic context, e.g. “the author writes the book” (expected condition), “the author writes the speech” (unexpected condition) or “the author writes the night” (anomalous condition). The sentence-final words in these three conditions were matched for frequency, word stress and number of letters and syllables. An additional condition with sentence-final pseudowords allowed for the implementation of a lexical decision task, i.e. to decide whether the final word is a pseudoword or a real word (indicated by button press). The advantage of using a lexical decision task is that semantic control processes are investigated implicitly and domain general executive requirements are kept stable across all conditions. The stimuli for our experiment were taken from a prior study from our laboratory [21]. In total 260 spoken sentences were auditorily presented via headphones (52 with expected, 52 with unexpected, 52 with anomalous and 104 with pseudoword endings). Additionally, subjects were presented 50 training sentences before the main experiment to familiarize with task and stimuli. The experiment was implemented in Presentation 14.9 (Neurobehavioral Systems). Trials were separated by random intertrial intervals between 1.5 and 4.5 s and presented in pseudorandomized order to make the condition of the current trial unpredictable.

### Statistical analysis

#### Behavioral data

Reaction times (RTs) and response correctness were extracted for each subject and each trial using Matlab R2016a (MathWorks). Incorrect trials and outliers (i.e. RTs greater than mean plus 2 standard deviations) were discarded and mean RTs were calculated for each subject and condition. Additionally, normalized mean unexpected, anomalous and pseudoword RTs were calculated by dividing the respective RTs by mean expected RTs. Further group level statistical analysis was conducted with IBM SPSS Statistics (version 24.0.0.0). All data were tested for normal distribution with Kolmogorov-Smirnov-Lilliefors-test. A 3x4 factorial repeated measures analysis of variance (ANOVA) of mean RTs with the factors stimulation site (aIFG, pMTG, sham) and condition (expected, unexpected, anomalous, pseudoword) was calculated. An additional 3x3 factorial repeated measures ANOVA was calculated for normalized RTs with the factors stimulation site (aIFG, pMTG, sham) and condition (unexpected, anomalous, pseudoword). Degrees of freedom were adjusted according to Greenhouser-Geisser whenever the assumption of sphericity was violated as indicated by the Mauchly-test (p < .05). Post-hoc paired t-tests (and Bonferroni correction) were applied if a factor or interaction explained a significant fraction of variance as indicated by the corresponding F-test.

Because error rates were not normally distributed, differences were assessed with direct pairwise comparisons conducting Wilcoxon signed rank tests for error rates pooled over (a) condition and (b) session respectively to assess main effects and with (c) unpooled data to check for cTBS effects (i.e. aIFG/pMTG stimulation vs. sham) within each condition. Bonferroni correction was used to control the family wise error rate.

#### Resting-state functional magnetic resonance imaging

Preprocessing and group level analysis were carried out using Statistical Parametric Mapping version 12 (SPM12 rev6685, Wellcome Trust Centre for Neuroimaging, London). Denoising, assessment of functional connectivity and statistical analysis of ROI-to-ROI connectivity was implemented in Matlab R2016a (MathWorks) with in-house scripts as outlined below.

The first four functional (EPI) scans were excluded from further analysis to allow for magnetic field saturation. Remaining scans were motion corrected using a two pass procedure with realignment (using a least squares approach and a rigid body spatial transformation with six degrees of freedom) of all scans to the first, calculation of a mean and realignment of all scans to the mean image. The structural (i.e. T1 weighted) image was coregistered (objective function: normalized mutual information) to the mean functional image and then segmented using the unified segmentation approach [53] with light bias regularization. This resulted in individual probability maps for grey/white matter and cerebrospinal fluid (CSF) and a nonlinear deformation field which was used to spatially normalize and resample (4^th^ degree B-spline interpolation to a voxel size of 3x3x3 mm) all functional scans to the MNI152 (Montreal Neurological Institute) space. To account for residual anatomical variance and to improve signal-to-noise ratio, all functional images were convolved with an isotropic Gaussian smoothing kernel with full width at half maximum of 8 mm.

For denoising purposes, BOLD signal variance over time explained by nuisance variables was removed from the data using a multiple regression approach. To this end, motion parameters (as derived from the realignment) and their first derivative (as first and second order terms) and mean white matter and CSF signal (as first order terms only) entered the regression model as explanatory variables. Mean white matter and CSF signal were extracted from individual tissue masks. To avoid bias introduced by grey matter signal regression, this signal was not included in the model. Furthermore, mean white matter signal was calculated only within areas with high (i.e. greater than .75) individual tissue probability [54]. Next, BOLD time series were band-pass filtered to preserve only frequencies between .01 and .08 Hz [22,23]. Because subject motion can disturb functional connectivity patterns, motion scrubbing was used to further improve data quality. This was achieved by calculation of framewise displacement (FD) defined as maximum frame to frame movement of any voxel within a 50 mm sphere centered in the sample volume [55]. All volumes with FD greater than .5 mm were discarded. This led to exclusion of one subject with excessive motion because remaining volumes represented less than 5 minutes of resting-state fMRI for each session. For the remaining 19 subjects, only 1.3% of all scans had to be discarded.

Regions of interest were defined as spheres with a radius of 7 mm around the peak coordinates derived from a prior fMRI study which—using the same task as in the present study—localized semantic control processes within the left aIFG, aMTG and pMTG [21] (see Fig 1B). These spheres were masked with individual grey matter masks to eliminate all voxels outside the brain and within white matter. BOLD time series for each ROI were then expressed as the first eigenvariate [56] of the time series of all remaining voxels within that ROI. Functional connectivity was calculated as Fisher transformed [57] Pearson correlation coefficient either between the time series of all possible pairs of ROIs (ROI-to-ROI) or between the ROI time series and the time series of all other voxels within the brain (whole brain). Whole brain functional connectivity on the group level was analyzed with a linear regression model at each voxel (mass univariate approach), using generalized least squares with a global repeated measures correlation model as implemented in SPM12. A repeated measures ANOVA with the factors stimulation site (aIFG, pMTG, sham), ROI (aIFG, aMTG, pMTG) and subject with appropriate non-sphericity correction was estimated. Contrasts of interests were connectivity after sham stimulation (for all three ROIs) and differences of these patterns induced by cTBS. These differences were examined by conjunction null analysis of the contrasts for ROI connectivity (after sham stimulation) and differences of this pattern between aIFG/pMTG and sham stimulation. Significance was assessed using cluster-level inference based on Gaussian random fields theory [58] as implemented in SPM12 with a cluster-forming threshold of p(uncorrected) < .001 and a significance threshold for cluster extent of p(FWE) < .05 [59]. Anatomical labeling was performed with the AAL2-toolbox (anatomical automatic labeling) for SPM12 [60,61]. To quantify lateral preferences, a laterality index (LI) was calculated for each subject and ROI (and for the conjunction of all three) as given by LI = (LH ‒ RH)/(LH + RH) based on the number of suprathreshold (p < .001) voxels in the left (LH) and right (RH) hemisphere [62]. Similarly to the whole brain analysis, network (ROI-to-ROI) connectivity after sham stimulation was assessed with one-sample t-tests and effects of cTBS with paired t-tests. To test for functional relevance of functional connectivity within this network, we calculated correlations between ROI-to-ROI connectivity after sham cTBS and normalized (unexpected and anomalous) RTs. Bonferroni correction was used to control the family wise error rate. Because ROI-to-ROI functional connectivity was not normally distributed for the connection between left aMTG and pMTG in the sham session as shown by Kolmogorov-Smirnov-Lilliefors test (p = .034), all affected statistical tests were instead conducted with appropriate non-parametric approaches (Spearman’s rank correlation and Wilcoxon signed-rank test). To finally test whether results are specific for the semantic control network, we tested for correlation with behavior and effects of cTBS on connectivity within a network not involved in semantic control, i.e. the default mode network (DMN). To this end, we obtained MNI coordinates of four DMN nodes from a prior resting-state fMRI independent component decomposition [63], i.e. medial frontal gyrus (6, 58, 4), posterior cingulate cortex (4, -52, 24), left angular gyrus (-50, -64, 30) and right angular gyrus (52–64 36) and tested whether connectivity between all possible pairs of DNM-ROIs is associated with behavior or modulated by cTBS.

## Results

### Behavioral data

**Fig 2:**
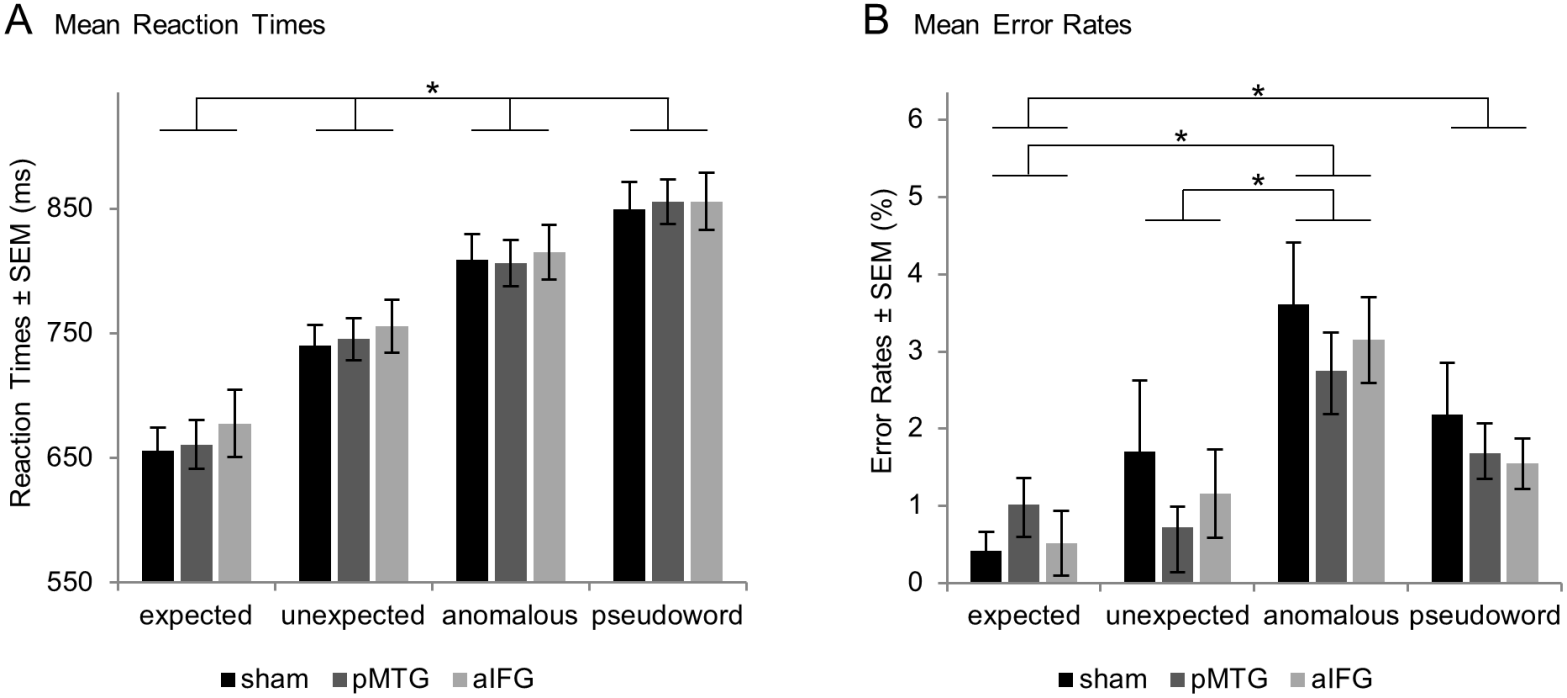
Behavioral results. (A) Mean reaction times increase with semantic control demands but do not differ between stimulation sites. (B) Error rates are highest for anomalous sentence endings without significant influence of stimulation site (n = 19, *p < .05, Bonferroni corrected).

Behavioral data were available for 19 subjects only due to technical issues with the response box. RTs in all conditions were normally distributed. Fig 2A displays raw mean RTs. Repeated measures ANOVA both for raw and normalized RTs revealed a significant main effect for condition (F-test: p < .001) but neither for stimulation site nor for their interaction (p > .05 respectively). Effects of condition were further analyzed with Bonferroni corrected post-hoc paired t-tests indicating significant differences between all possible pairs of conditions both for raw and normalized RTs revealing a pattern of monotonously increasing RTs over the four conditions (expected < unexpected < anomalous < pseudoword).

Error rates were small with an overall mean of 1.7% (corresponding to about 5 of the total 260 trials per session, see Fig 2B) and showed a non-normal distribution. Non-parametric testing (Bonferroni corrected Wilcoxon signed rank tests) revealed the following significant differences between conditions: expected < anomalous, expected < pseudoword and unexpected < anomalous. This suggests an analogous pattern compared to the RTs except for pseudowords which led to better accuracy compared to anomalous endings. No significant differences were found between stimulation sites neither across all nor within each condition (p > .05 respectively).

### Functional connectivity

**Fig 3:**
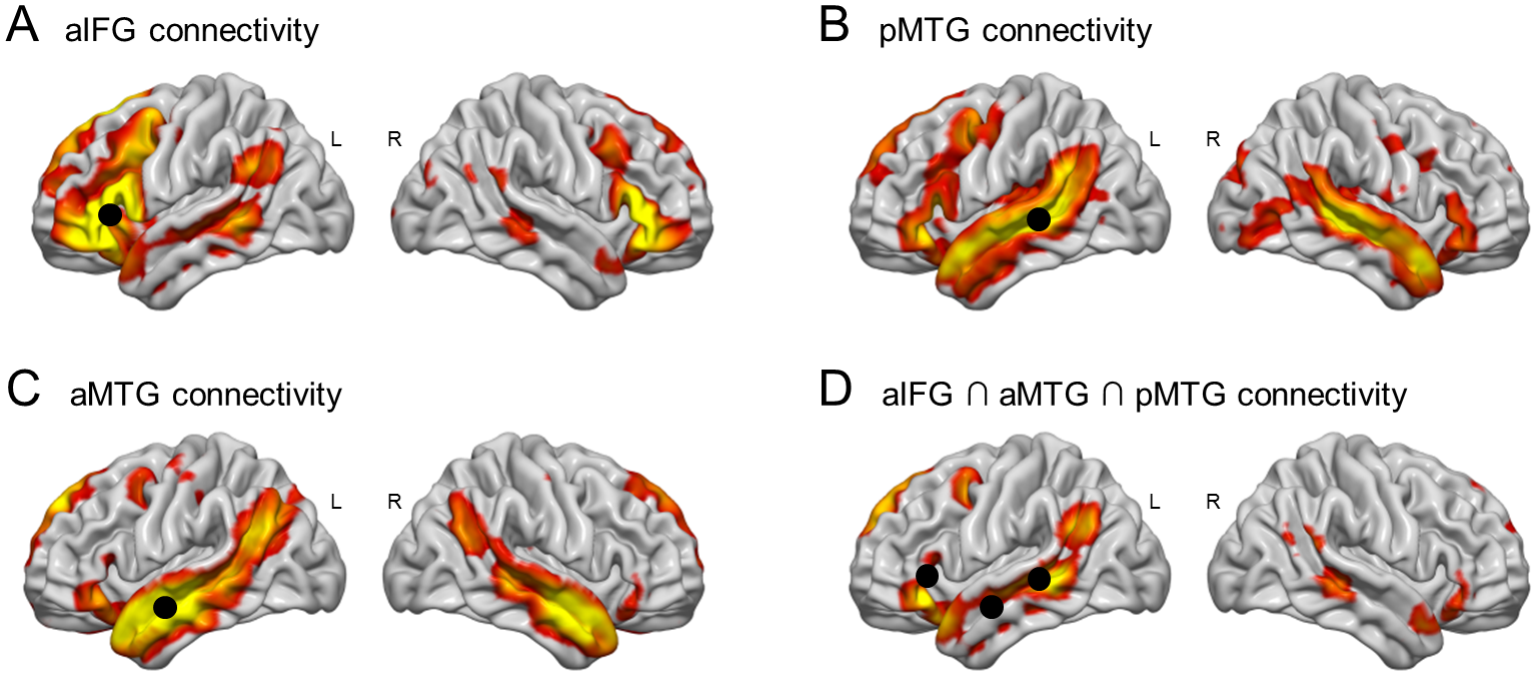
Whole brain resting-state functional connectivity of aIFG, aMTG and pMTG. Resting-state functional connectivity of left (A) aIFG, (B) pMTG, (C) aMTG and (D) their overlap of 19 healthy subjects after sham continuous theta burst stimulation. Panels (A)-(C) show a left-lateralized but rather unspecific pattern, while (D) reveals a specific left-lateralized fronto-temporal network corresponding to known semantic nodes. Renderings are based on thresholded T-maps with p(FWE) < .05 on the cluster-level and a cluster-forming threshold of p < .001 (uncorrected). Black circles indicate regions of interest.

Whole brain resting-state functional connectivity of left aIFG, aMTG and pMTG after sham cTBS revealed widespread, predominantly fronto-temporal and slightly left-lateralized (mean LI: aIFG: 0.16, aMTG: 0.08 and pMTG: 0.09) networks (see Fig 3 and S1 Table) with strongest functional connectivity to the homologous area in the right hemisphere. The overlap (i.e. conjunction null analysis) of all three ROIs revealed a significantly more left-lateralized (LI of 0.34) fronto-temporal pattern including bilateral inferior frontal, superior and middle temporal, angular (and supramarginal) gyri and temporal poles as well as left middle frontal and medial superior frontal gyri.

Additional functional connectivity of the aIFG includes subcortical structures such as left caudate nucleus, pallidum and putamen and also the right cerebellum. Connectivity of aMTG also included a bilateral postero-medial cluster containing lingual gyrus, precuneus and middle/posterior cingulum extending bilaterally to thalamus and hippocampus as well as an antero-medial cluster including medial superior frontal gyri, medial frontal cortices and straight gyri. Connectivity of pMTG additionally comprised bilateral precentral gyri, precuneus and cuneus, calcarine sulcus, lingual gyri and occipital cortex as well as right fusiform gyrus. For a complete list of anatomical labels see S1 Table. Although all ROIs were defined in the left hemisphere and left lateralization is therefore not surprising, it is worth noting that the LI was significantly greater (as shown by paired t-test) for the overlap than for the single ROIs.

Effects of cTBS on whole brain connectivity of different ROIs were tested by conjunction null analysis of contrasts for ROI connectivity (after sham stimulation) and differences of this pattern between stimulation over aIFG/pMTG and sham stimulation. This analysis did not reveal any significant effects of cTBS on ROI connectivity. Even when inspected at a threshold of p < .001 on voxel-level without any correction for multiple comparisons, no compelling spatial pattern arose. Contrary, contrasts of interest revealed either empty maps or unstructured noise to become manifest in very small clusters (with corresponding p(FWE) > .35) at apparently random positions. One could argue that effects of cTBS could also appear in regions which had no significant connectivity to a certain ROI after sham stimulation (e.g. regions which are recruited due to cTBS). We, therefore, repeated our analysis without conjunction to avoid bias introduced by a priori spatial limitation of differential effects to certain regions. However, this also did not provide evidence for significant influence of cTBS on ROI connectivity.

**Fig 4:**
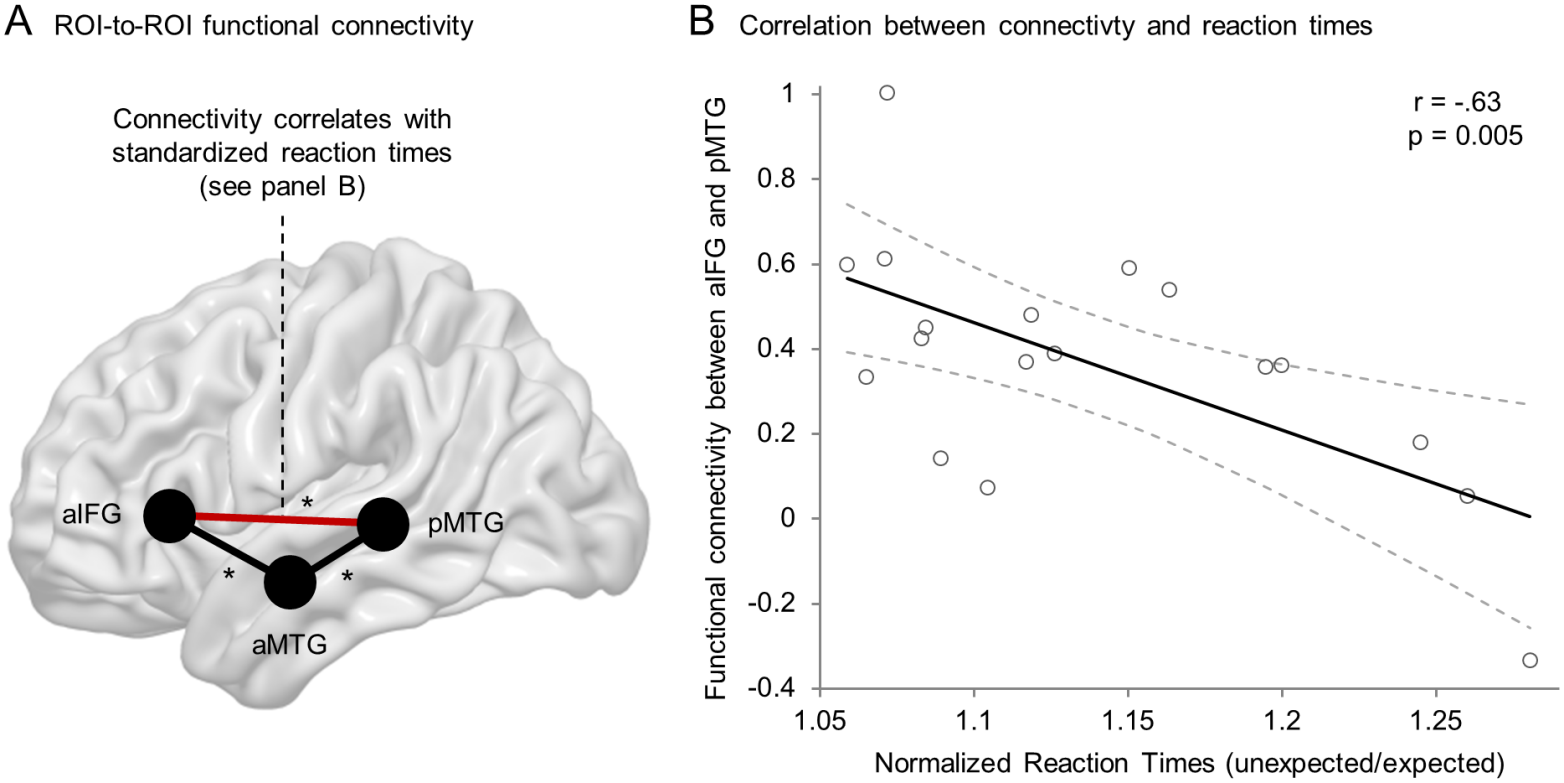
ROI-to-ROI functional connectivity and its correlation with reaction times. (A) ROI-to-ROI resting-state functional connectivity of 19 healthy subjects after sham continuous theta burst stimulation reveal significant connectivity between all pairs of ROIs (*p < .05, Bonferroni corrected). (B) Linear correlation analysis reveals higher resting-state functional connectivity (after sham cTBS) between aIFG and pMTG (shown in red in panel A) to be associated with faster normalized reaction times for unexpected sentences (n = 18, p < .05, Bonferroni corrected). Solid line matches best linear fit with 95% confidence interval indicated by dashed lines.

As whole brain connectivity patterns suggested, ROI-to-ROI connectivity between all pairs of ROIs within the semantic control network was significantly (Bonferroni corrected) larger than zero for the sham session (Fig 4A). However, again ROI-to-ROI connectivity could not reveal any significant influence of cTBS on functional connectivity neither within the semantic network nor within the DMN (p > .05, uncorrected).

### Correlation between connectivity and behavioral data

Because raw RTs are rather unspecific and also depend on processes outside the language domain (i.e. perceptive, executive and motor functions), we decided to control for these unspecific effects by contrasting all semantic decisions with high control demands (i.e. sentences with unexpected/anomalous endings) with sentences with low control demands (i.e. sentences with expected endings). This was done by normalizing RTs in trials with unexpected and anomalous endings to RTs in trials with expected endings. The rationale followed a similar strategy aiming to eliminate unspecific neural activity in a previous fMRI study [21]. Both behavioral and functional connectivity data were available for 18 subjects. Using data obtained in the sham condition correlation analysis was performed between normalized RTs in high control demand trials and connectivity between all ROI pairs (Table 1). This analysis revealed that higher functional connectivity between left aIFG and pMTG was significantly (Bonferroni corrected) associated with faster response times for unexpected stimuli (see Fig 4B). There was also a trend towards an association of faster response times for anomalous stimuli with higher connectivity for this connection (p = .01), which however did not survive Bonferroni correction. Because one might argue that these correlations could be driven by the dividend or divisor of the normalized RTs, it is worth noting that neither of these alone significantly correlated with the connectivity measure. Furthermore, these associations between behavior and connectivity were specific for the semantic control network and not evident for connectivity between any pair of four DMN nodes (p > .05, uncorrected).

**Table 1:** Correlations between functional connectivity and behavior.

	Resting-state functional connectivity		
	aIFG-aMTG	aIFG-pMTG	aMTG-pMTG
**Normalized reaction times (unexpected/expected)**	r = -.46	r = -.63*	r = .03
	p = .055	p = .005	p = .895
**Normalized reaction times (anomalous/expected)**	r = -.37	r = -.59	r = .12
	p = .136	p = .010	p = .638

Correlation analysis between normalized reaction times in high control demand trials and connectivity between all pairs of ROIs after sham continuous theta burst stimulation (n = 18, *p < .05, Bonferroni corrected).

Finally, we performed an additional exploratory analysis to test whether the described correlation found between connectivity between aIFG and pMTG and behavior in the sham condition (r = -.63) was significantly different after stimulation over aIFG (r = -.17) or pMTG (r = -.42), respectively. To this end, we performed a two-tailed permutation test with the null hypothesis that the correlation coefficient is not dependent on the cTBS condition for both aIFG and pMTG vs. sham. This was achieved by 10,000 random assignments of cTBS condition (real vs. sham) to individual data followed by computation of the difference of Fisher transformed [57] correlation coefficients to obtain the null distribution. We found that the correlation of connectivity between aIFG and pMTG and normalized unexpected RTs in the sham condition was significantly weaker after cTBS over aIFG (p = 0.02) but was not different after cTBS over pMTG (p = 0.19).

## Discussion

In the present study, we used functional connectivity measures based on slow intrinsic BOLD signal fluctuations during wakeful rest to characterize interactions between key nodes of the semantic network. Connectivity patterns of regions of interest in left aIFG, pMTG and aMTG are in accordance with the semantic network as known from previous task-based fMRI studies. Importantly, we found evidence for the functional relevance of the interaction between left aIFG and pMTG at rest for semantic control. This was based on a correlation with performance in a semantic task for trials with high semantic control demands. However, we did not obtain evidence for modulating effects of cTBS applied over semantic key regions.

To examine the functional relevance of resting-state functional connectivity within the semantic network, we correlated behavioral measures from a well-established semantic priming paradigm [21] with connectivity measures between our predefined ROIs in the sham condition. We showed that functional connectivity between left aIFG and pMTG is relevant for semantic control processes on sentence level. Specifically, higher connectivity between these two nodes was associated with faster integration of the final key word under high semantic control demands. The direction of this association, i.e. higher connectivity correlates with better performance is typically observed for cognitive processing [32]. Our finding is well in line with a prior study by Wei and colleagues who showed that functional connectivity strength between aIFG and pMTG during rest was associated with general semantic performance, although this association did not further explain performance scores when regional BOLD signal amplitudes in pMTG were regressed out [28]. Probabilistic fiber tracking has revealed the most probable anatomical substrates underlying this functional interaction: (i) a ventral pathway consisting of the inferior fronto-occipital fasciculus (iFOF) running through the extreme capsule and (ii) a dorsal pathway consisting of the superior longitudinal fasciculus/arcuate fasciculus (SLF/AF) [21]. The ventral pathway is indeed associated with semantic processing [64,65] and therefore the most likely structural correlate of the observed association between functional connectivity and performance in the semantic priming paradigm. Recent analyses of effective connectivity in task-based fMRI using dynamic causal modeling (DCM) suggest that higher semantic control demands require inhibition of the left pMTG in order to suppress the dominant meaning and that the source of this inhibition is either the aMTG or the aIFG depending on control demands [21]. We can only speculate, whether the discovered association between functional connectivity at rest and performance reflects the ability of left aIFG to dynamically inhibit left pMTG during task performance. If this were the case, our data would not support the assumption of multiple (i.e. aMTG → pMTG and aIFG → pMTG) inhibitory routes depending on semantic control demands, because we only observed a correlation between behavior and aIFG-pMTG connectivity for unexpected and (as a statistical trend) anomalous sentences, but no correlation for the connectivity between aMTG and pMTG. Alternatively, this association between interactions during rest and behavior could also reflect other (unknown) mechanisms which contribute differently to semantic performance. In either case, our findings, using a method complementary to DCM on fMRI data, support the view that a functional interaction between aIFG and pMTG might represent the neural correlate of semantic integration processes under high control demands.

One could argue that the association between behavior and connectivity is fixed (e.g. structurally determined) [32]. It is thus of interest to prove that connectivity is context-dependent. We, therefore, aimed to modulate specific connectivity within the semantic network by application of cTBS over semantic key regions (i.e. aIFG and pMTG). However, we did not find any direct modulating effects of cTBS, neither on behavior nor on resting-state functional connectivity. We therefore, tested whether effective cTBS affected the correlation found for connectivity between left aIFG and pMTG and behavioral performance. Indeed, this analysis revealed that the association between aIFG-pMTG connectivity and behavior was significantly reduced after real cTBS over aIFG but not over pMTG. We interpret this as an indirect disruptive effect of cTBS over aIFG. That is, interactions between left aIFG and pMTG are functionally less relevant for semantic control after temporary disruption of aIFG compared to sham stimulation while behavioral performance remains unaffected. The absence of a behavioral effect might be explained by (unobserved) functional reorganization within the network fully compensating the focal disturbance induced by cTBS over aIFG. Additionally, functional degeneracy [66] in the semantic network might protect the system against unifocal disturbance. This is in line with a recent TMS-study that aimed at modulating semantic processing and showed that inhibition of a single network node was insufficient to delay reaction times in a semantic decision task [67].

Although this indirect effect of cTBS on semantic processing certainly is of interest, the absence of a direct stimulation effect on connectivity or behavior needs to be discussed. This might be explained by (i) the resilience of resting-state functional connectivity in general, (ii) insufficient effectivity of the cTBS protocol and (iii) limitations of our study design to detect cTBS effects. The first possibility seems to be unlikely because single session cTBS has already been shown to alter resting-state functional connectivity in healthy subjects when applied over temporal [47], parietal [41], occipital [41] and frontal [44] regions and has also been proven to be suitable to disrupt behavior in the cognitive domain [68,69].

Regarding the stimulation intensities a previous study showed that cTBS over temporal cortex only affected lexical decisions when applied at a higher stimulation intensity of 90% of individual AMT, but not with the conventional protocol of 80% AMT as used in our study [70]. Insufficient magnetic field strengths at the stimulation target might have been further amplified by larger coil to cortex distances of the stimulation targets. This might be in particular true for the pMTG with additional 1.0–1.5 cm when compared to the motor cortex which was used to determine individual stimulation intensities [71]. Concerning the study design, the implementation of a single resting-state fMRI session after administration of cTBS [40,72,38,41] might have rendered our findings vulnerable against effects induced by the passage of time. Although resting-state networks are known to be stable over time [73], intrasubject variability between two sessions may be larger than TMS induced effects on connectivity [45]. It is thus likely that pre- and post-TMS resting-state fMRI designs are more sensitive (although less specific) to TMS induced effects. As inter-session interval amounted to at least one week in our study, performing pre- as well as post-TMS scanning sessions, as done in several previous studies [45,42,44,39,43,47,46], might have been able to reduce inter-session variability. Another concern is related to the timing of our resting-state session, as cTBS effects on the BOLD signal are strongly time-dependent. Resting-state scanning in our study started about 9 minutes after stimulation and lasted 6 minutes. It has recently been shown for a saccade-fixation paradigm that effects on task-related activity do not arise before 20 to 35 minutes after cTBS [74]. Similar results were also obtained in resting-state fMRI: Gratton and colleagues observed increased connectivity in widespread networks after cTBS over cognitive control areas 20 minutes but not 10 minutes after stimulation [44]. Since functional connectivity within a given network may be stronger during task performance it can be speculated that task-related connectivity is more sensitive to cTBS effects.

Resting-state functional connectivity of regions of interest in left IFG, pMTG and aMTG revealed highly interconnected left-lateralized fronto-temporal networks representing the semantic system. All three ROIs were associated with semantic control processes in prior task-based fMRI studies [21,20]. Each of these ROIs revealed a widespread and highly interconnected, mostly fronto-temporal network in accordance with prior resting-state studies [75,35,37]. These connectivity patterns overlapped in strongly left-lateralized regions displaying a striking similarity to activation patterns found in previous task-based semantic fMRI studies [76]. This set of interconnected nodes features regions which are involved in specific semantic tasks. This includes regions which may serve as an amodal semantic store, i.e. parts of the anterior temporal lobe [3] and regions which have been suggested to be involved in sematic control processes like parts of the posterior middle temporal gyrus [2,7,1], the left dorsomedial prefrontal cortex [7,76], the pars orbitalis of the inferior frontal gyrus [2,7,1] which has also been suggested to be involved in semantic unification of multi-word utterances [14]. Connectivity networks also overlapped in the angular gyrus (extending slightly to the supramarginal gyrus). The exact role of this region for semantic cognition remains elusive. It may be directly involved in semantic control [7] or automatic semantic retrieval [76,77] and contribute indirectly to semantic cognition due to its involvement in more domain-general executive processing [78]. These observations support the view that slow intrinsic BOLD signal fluctuations during rest in combination with our predefined ROIs reveal a network of interconnected regions which highly corresponds to the semantic system. This might suggest that functional connectivity within this network coordinates neuronal processing and therefore facilitates the observed co-activations in semantic fMRI paradigms.

Functional connectivity of aIFG to left basal ganglia and the right cerebellum is also in accordance with prior resting-state connectivity analyses [35,75] and supports a possible role of these regions in language processing. While the importance of the right cerebellum in language processing has long been discussed [79], there are also recent studies which link the basal ganglia to synchronization of temporal and sequential aspects in language processing [80].

On the behavioral level, performance reflected the increasing semantic control demands for unexpected and anomalous compared to expected sentence endings, indicating that semantic incongruencies between the sentence’s subject and the lexical decision target (unexpected condition) disturb the lexical decision response. This disturbance was even stronger for additional incongruencies between the verb and the target (anomalous condition). These findings are well in line with prior findings on the same task both for visual [20] and auditory [21] stimulus presentation and confirm that it appropriately represents semantic integration processes under increasing semantic control demands.

The ROIs and stimulation targets used in this experiment were taken from a previous study utilizing the same task as in the current work [21]. These ROIs therefore correspond to regions specifically involved in neural processing associated with this particular task and are well suited especially for the correlation with behavioral data obtained in this study. Nevertheless, it has to be noted that the coordinates of these ROIs differ to some degree from meta-analytic data regarding semantic control networks. For example, the pMTG coordinate used in this study is located more ventrally than the peak from an activation likelihood estimation analysis of studies contrasting high and low semantic control [7]. Our results might thus be specific for semantic control processes considering the given task but less generalizable to this process when using different tasks.

## Conclusions

Using resting-state fMRI, we were able to reproduce the semantic network as known from previous task-based fMRI studies. This indicates that functional connectivity within this network might coordinate neuronal processing and therefore facilitate the observed co-activations in semantic fMRI paradigms. Our main finding was an association between resting-state functional connectivity between aIFG and pMTG and task performance suggesting that a functional interaction between aIFG and pMTG might represent the neural correlate of semantic integration processes under high control demands. This interaction was functionally less relevant after cTBS over aIFG which is indicative for an indirect disruptive effect of cTBS on semantic control. However, this study did not reveal any significant direct effects of cTBS on semantic task performance or functional connectivity which might be explained by experimental limitations. Future studies should consider higher stimulation intensities for cTBS, longer intervals between cTBS and subsequent resting-state fMRI and additional baseline scanning prior to the intervention in order to optimize the chance to detect direct behavioral and neurobiological effects of cTBS.

## Supporting information

S1 Table Whole brain resting-state functional connectivity of aIFG, aMTG and pMTG. Resting-state functional connectivity of left aIFG, pMTG aMTG and their overlap of 19 healthy subjects after sham continuous theta burst stimulation. Significance threshold: p(FWE) < .05 on cluster-level with a cluster-forming threshold of p(uncorrected) < .001 on voxel-level. Anatomical labels according to Anatomical Automatic Labeling 2 (AAL2) for SPM12 where % label was > 5%. Cluster extent is noted in milliliters. Peak level coordinates (reporting up to 3 per cluster) refer to Montreal Neurological Institute (MNI) space. Abbreviations: aIFG—left anterior inferior frontal gyrus, aMTG—left anterior middle temporal gyrus, pMTG—left posterior middle temporal gyrus.
(PDF)
