# Supplementary material for: Fronto-temporal interactions are functionally relevant for semantic control in language processing
Source: PLoS One. 2017 May 15;12(5):e0177753. doi: 10.1371/journal.pone.0177753 (PMC5432178; doi:10.1371/journal.pone.0177753)
Supplement: S1 Table — Resting-state functional connectivity of left aIFG, pMTG aMTG and their overlap of 19 healthy subjects after sham continuous theta burst stimulation. Significance threshold: p(FWE) < .05 on cluster-level with a cluster-forming threshold of p(uncorrected) < .001 on voxel-level. Anatomical labels according to Anatomical Automatic Labeling 2 (AAL2) for SPM12 where % label was > 5%. Cluster extent is noted in milliliters. Peak level coordinates (reporting up to 3 per cluster) refer to Montreal Neurological Institute (MNI) space. Abbreviations: aIFG—left anterior inferior frontal gyrus, aMTG—left anterior middle temporal gyrus, pMTG—left posterior middle temporal gyrus. (PDF) [file pone.0177753.s001.pdf]

S1 Table. Whole brain resting-state functional connectivity of aIFG, aMTG and pMTG. Resting-state functional connectivity of left aIFG, pMTG aMTG and their overlap of 19 healthy subjects after sham continuous theta burst stimulation. Significance threshold: p(FWE) < .05 on cluster-level with a cluster-forming threshold of p(uncorrected) < .001 on voxel-level. Anatomical labels according to Anatomical Automatic Labeling 2 (AAL2) for SPM12 where % label was > 5 %. Cluster extent is noted in milliliters. Peak level coordinates (reporting up to 3 per cluster) refer to Montreal Neurological Institute (MNI) space. Abbreviations: aIFG – left anterior inferior frontal gyrus, aMTG – left anterior middle temporal gyrus, pMTG – left posterior middle temporal gyrus.

**cluster-level**			**peak-level**	
**labeling according to AAL2-toolbox for SPM12**	**p(FWE)**	**extent**	**T_{144}**	**x, y ,z (mm)**
**aIFG functional connectivity**				
Frontal_Inf_Oper_L, Temporal_Pole_Mid_L, Frontal_Inf_Orb_2_L, Frontal_Sup_Medial_R, Frontal_Sup_2_R, Precentral_L, Frontal_Sup_2_L, OFCant_L, Rolandic_Oper_L, Supp_Motor_Area_R, Frontal_Inf_Tri_L, Temporal_Mid_L, Caudate_L, Cingulate_Ant_L, Temporal_Pole_Sup_L, SupraMarginal_L, Angular_L, Frontal_Mid_2_L, OFClat_L, Frontal_Sup_Medial_L, Putamen_L, Pallidum_L, Insula_L	0,000	168,7	38,3	-51, 26, 2
			10,9	-6, 20, 62
			10,6	-9, 41, 44
Frontal_Inf_Orb_2_R, Frontal_Inf_Oper_R, OFCant_R, Frontal_Inf_Tri_R, Temporal_Pole_Mid_R, OFClat_R, Insula_R, Temporal_Pole_Sup_R, OFCpost_R	0,000	37,7	14,2	57, 29, 2
			6,2	48, 11, 44
			5,7	45, 14, -22
Temporal_Inf_R, Temporal_Mid_R	0,000	9,9	5,6	66, -49, 20
			5,4	63, -31, -7
			4,9	54, -34, -1
Cerebelum_Crus2_R, Cerebelum_6_R, Cerebelum_7b_R	0,004	3,2	4,3	27, -76, -43
			4,2	15, -79, -34
			4,2	24, -70, -31
**aMTG functional connectivity**				
Temporal_Mid_R, Frontal_Inf_Tri_L, Temporal_Pole_Mid_L, Temporal_Pole_Sup_L, Precuneus_R, Thalamus_L, Angular_R, Temporal_Sup_R, Temporal_Inf_R, Precuneus_L, Temporal_Pole_Sup_R, Temporal_Inf_L, Temporal_Mid_L, Calcarine_R, Frontal_Inf_Orb_2_R, Cuneus_R, Lingual_R, Hippocampus_R, Cerebelum_4_5_L, Temporal_Pole_Mid_R, Amygdala_L, Fusiform_R, Parietal_Sup_L, Occipital_Sup_L, Cuneus_L, Cingulate_Post_R, Cingulate_Mid_R, Parietal_Inf_R, ParaHippocampal_L, Frontal_Inf_Tri_R, SupraMarginal_R, ParaHippocampal_R, Lingual_L, OFClat_L, Hippocampus_L, Cingulate_Post_L, Olfactory_L, Amygdala_R, OFCpost_R, Occipital_Mid_R, Parietal_Sup_R, Insula_R, Angular_L, Occipital_Sup_R, Frontal_Inf_Orb_2_L, OFClat_R, Thalamus_R	0,000	233,2	28,9	-54, -7, -16
			12,5	57, -1, -19
			11,9	57, -10, -13
Frontal_Sup_Medial_R, Frontal_Med_Orb_L, Frontal_Sup_2_R, Rectus_R, Frontal_Sup_2_L, Frontal_Med_Orb_R, Frontal_Mid_2_L, OFCmed_L, Rectus_L, Cingulate_Ant_R, Cingulate_Mid_R, Olfactory_R, Olfactory_L	0,000	69,9	9,6	-3, 50, -16
			9,2	-12, 47, 44
			6,8	6, 50, 26
Frontal_Inf_Oper_L, Frontal_Mid_2_L	0,000	4,7	5,6	-36, 5, 50
Postcentral_L	0,000	6,8	4,8	-45, -22, 44

			4,1	-36, -16, 59
			4,0	-51, -19, 56
Postcentral_R	0,018	2,3	4,0	39, -7, 38
			3,8	54, -13, 56
			3,8	51, -13, 38
**pMTG functional connectivity**				
Temporal_Mid_R, Temporal_Pole_Sup_L, Temporal_Pole_Mid_L, Precentral_L, Temporal_Sup_R, Frontal_Mid_2_L, Frontal_Inf_Tri_R, Occipital_Sup_L, Lingual_R, Postcentral_R, Postcentral_L, Lingual_L, Cuneus_L, Temporal_Pole_Sup_R, Calcarine_R, Frontal_Inf_Orb_2_R, Temporal_Mid_L, SupraMarginal_R, Frontal_Inf_Oper_L, Temporal_Inf_L, Angular_R, Rolandic_Oper_R, Fusiform_L, Temporal_Inf_R, Occipital_Mid_L, Rolandic_Oper_L, Insula_R, Frontal_Inf_Orb_2_L, Frontal_Inf_Tri_L, Occipital_Inf_L, Cerebelum_4_5_R, Temporal_Pole_Mid_R, Precuneus_L, Angular_L, OFClat_L, Parietal_Inf_R, Occipital_Mid_R, Temporal_Sup_L, Heschl_R, OFCpost_R, OFCpost_L, Cuneus_R, Occipital_Inf_R, ParaHippocampal_L, OFClat_R, Heschl_L	0,000	251,8	27,8	-51, -31, 2
			13,2	48, -34, -1
			10,0	-54, 8, -16
Frontal_Sup_Medial_R, Frontal_Mid_2_L, Supp_Motor_Area_R, Cingulate_Ant_L	0,000	25,7	6,7	-6, 17, 62
			6,6	-9, 32, 56
			6,1	-15, 53, 32
Frontal_Inf_Tri_R, Frontal_Inf_Oper_R	0,046	1,8	5,1	36, 11, 32
Precuneus_R, Cingulate_Post_R, Cingulate_Post_L	0,000	7,0	5,1	-9, -52, 44
			5,1	-9, -49, 32
			4,6	6, -55, 41
Frontal_Sup_2_R	0,000	8,4	4,9	30, -19, 53
			4,3	60, -7, 44
			3,8	33, -28, 59
Supp_Motor_Area_R, Paracentral_Lobule_R	0,000	4,8	4,7	-6, -13, 41
			4,4	-6, -22, 50
			4,1	3, -22, 56
**aIFG, aMTG and aIFG functional connectivity (conj. null)**				
Temporal_Pole_Mid_L, Temporal_Mid_L, OFCpost_L, Temporal_Pole_Sup_L, Temporal_Sup_L, Frontal_Inf_Tri_L, Frontal_Inf_Orb_2_L, Angular_L, OFClat_L, SupraMarginal_L	0,000	45,0	7,8	-54, -43, 2
			7,6	-60, -31, -4
			7,4	-45, 29, -10
Frontal_Sup_Medial_R, Frontal_Mid_2_L, Cingulate_Ant_L	0,000	17,9	6,2	-9, 35, 53
			6,1	-12, 47, 44
			5,9	-12, 53, 29
Temporal_Pole_Mid_R, OFCpost_R, OFClat_R, Frontal_Inf_Orb_2_R	0,000	6,2	5,7	45, 14, -22
			4,5	45, 29, -16
			4,5	45, 32, -7
Frontal_Inf_Oper_L, Frontal_Mid_2_L	0,001	4,1	5,6	-36, 5, 50
Temporal_Mid_R	0,000	8,6	5,5	63, -52, 20
			5,4	63, -31, -7
			4,9	54, -34, -1
